# Supplementary material for: Precapillary sphincters maintain perfusion in the cerebral cortex
Source: Nat Commun. 2020 Jan 20;11:395. doi: 10.1038/s41467-020-14330-z (PMC6971292; doi:10.1038/s41467-020-14330-z)
Supplement: Supplementary file 1 — Supplementary Information [file 41467_2020_14330_MOESM1_ESM.pdf]

Supplementary Information

Precapillary sphincters maintain perfusion in the cerebral cortex

Grubb, Cai and Hald et al.

Supplementary Tables

Supplementary table 1 – Whisker-stimulated vasodilation

	PA (n=19, N=13)	Sphincter (n=19, N=13)	Bulb (n=11, N=9)	1st order capillary (n=18, N=12)
**Baseline diameter (µm)**	18.43±1.57	4.55±0.37	7.90±0.64	6.48±0.63
**Max vasodilation (%)**	10.97±2.27	33.75±4.08	12.92±3.98	15.53±2.27
**Max undershoot (%)**	-2.08±0.85	-12.40±2.10	-8.48±3.22	-6.71±2.45

Data presented as mean ± s.e.m. N = animals, n = vessels. See Fig. 3 for statistics. Source data are provided as a Source Data file.

Supplementary Figures

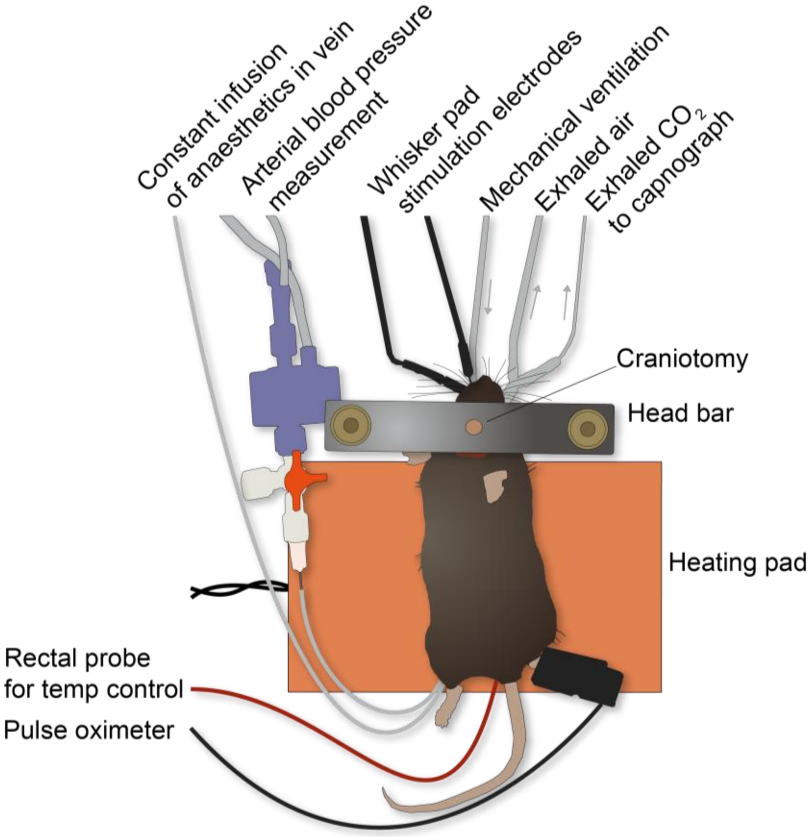

Supplementary Figure 1 | Illustration of the in vivo setup. The mouse was mechanically ventilated, anaesthetized with α-chloralose infusion into the left femoral vein (only after surgery), and kept warm using a heating pad with a rectal probe as reference. To monitor the physiological state of the animal we measured exhaled CO_2 with a capnograph, measured the left femoral artery blood pressure with a blood pressure monitor, and measured the oxygen saturation with a pulse oximeter. We also took blood samples to check the blood gasses and corrected ventilation and/or oxygen saturation of the mixed air to keep the animal in a physiological state. The mouse skull was glued to a head bar and a 3-mm craniotomy drilled over the right barrel cortex. Stimulation electrodes were inserted into the contralateral ramus infraorbitalis of the trigeminal nerve.

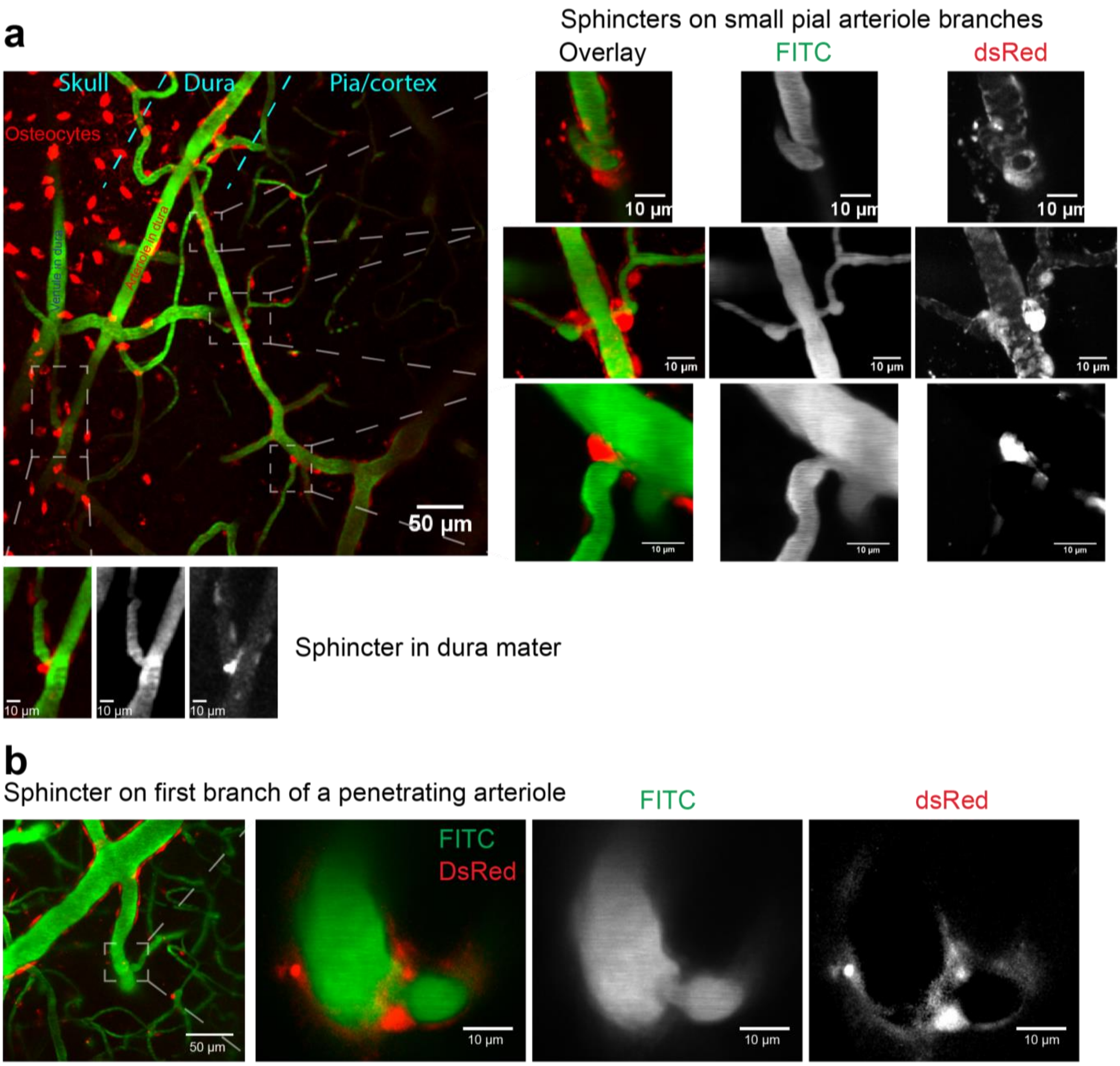

Supplementary Figure 2 | Two-photon imaging through thinned skulls of dsRed mice injected with FITC. ***a***, ***Left panel****: Example of a maximum intensity projection of a thinned skull experiment. The dsRed-positive osteocytes of the skull are seen on the left side (the image is taken on an angle, see Supplementary video 1), fading to the right into the blood vessels in the dura, and then to the far right fading into a pial arteriole and cortical capillaries.* ***Right panels****: Local projections of three precapillary sphincters on surface vessels (see also Supplementary video 1 and 4).* ***Lower panels****: Example of a precapillary sphincter on a dural arteriole branch.* ***b****, Another example of a thinned skull experiment, showing a maximum intensity projection of a penetrating arteriole, and on the right a local projection of the precapillary sphincter at the first branch.*

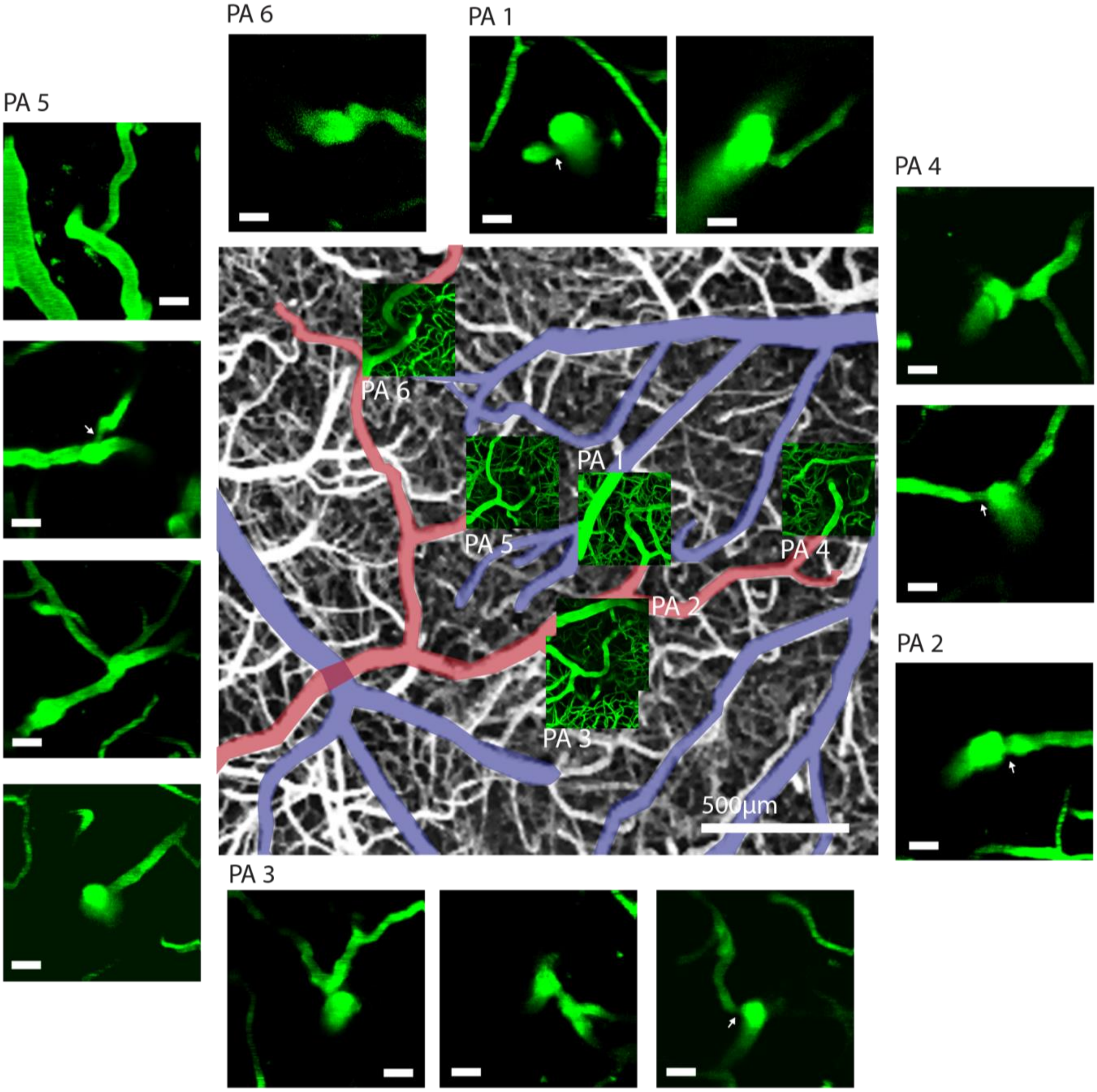

Supplementary Figure 3 | ***Middle****: Part of the whisker barrel cortex in a mouse with a C57bl6/j background. Vessels have been loaded with FITC-dextran. Veins have been coloured blue and arteries red. The maximum intensity projection of the uppermost 300 µm of the cortex surrounding six penetrating arterioles is shown.* ***Surrounding****: Maximum intensity projections of local z-stacks of branch points on the six penetrating arterioles are shown. The first row contains the uppermost branch points, the second row the second branch point, and so on. The scale bars are 20 µm. White arrows mark precapillary sphincters, but the precapillary sphincters on bifurcating PA branches are not marked.*

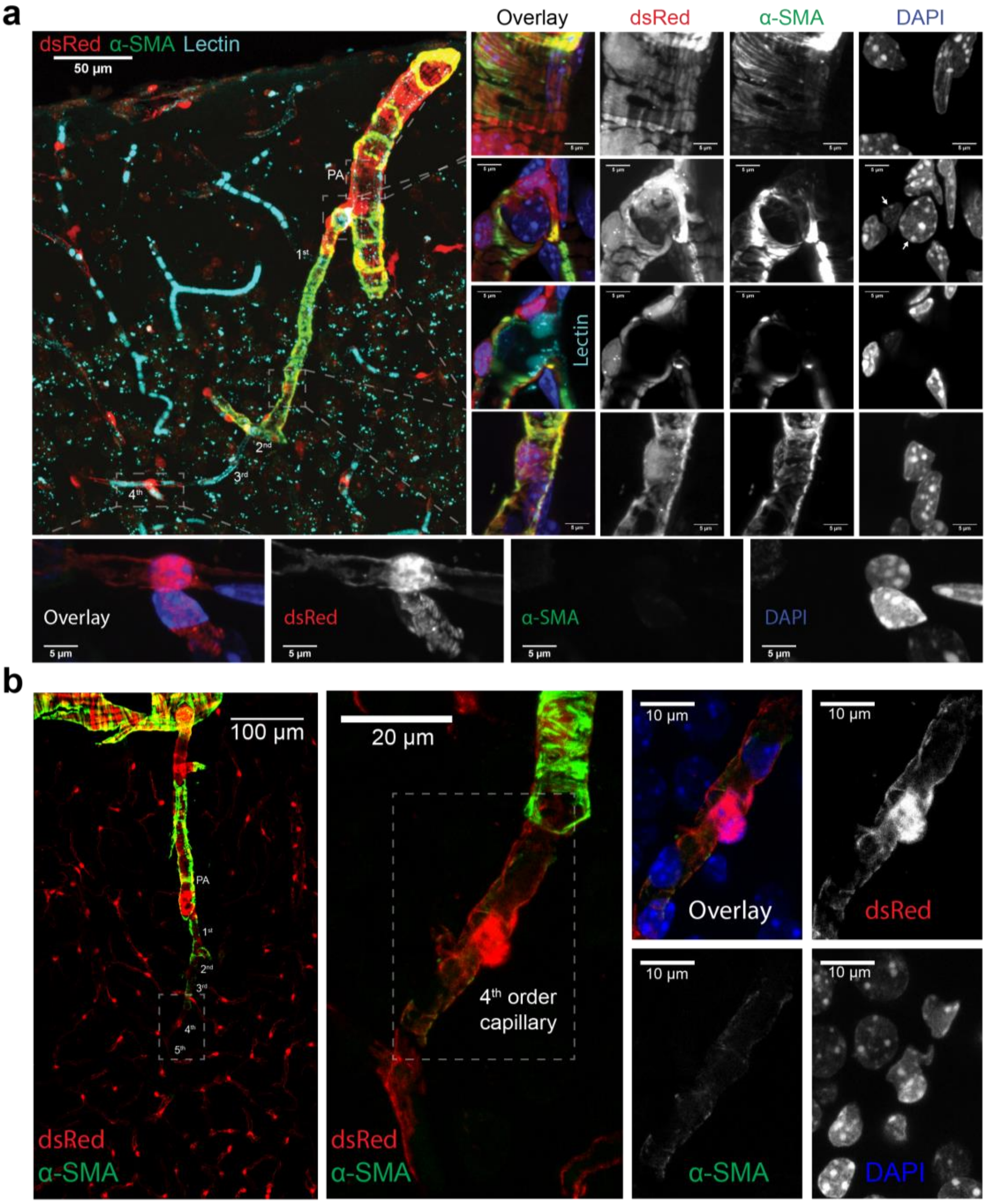

Supplementary Figure 4 | Immunostaining for α-smooth muscle actin in coronal slices of NG2-dsRed mice. ***a***, ***Top left***: *Maximum projection image of a penetrating arteriole in a mouse injected with tomato-lectin (cyan) 20 min before PFA fixation. Note that the tomato-lectin also stains residual RBCs in the capillaries.* ***Top right***: *Local projections of the PA are filamentous for α-SMA in VSMC/pericyte hybrids. In the second row, the precapillary sphincter exhibits α-SMA expression in the sphincter cell, an uncovered endothelial nucleus at the bulb, and α-SMA expression in the 1st order pericytes. In the third row down, a narrow projection (cross section) of the precapillary sphincter includes lectin staining, showing the narrow lumen of the precapillary sphincter and the large volume of the bulb. In the bottom row is a maximum projection of the far end of the 1st order capillary.* ***Lower panel***: *Maximum intensity projection of a 4th order capillary showing no α-SMA expression.* ***b***, *Another example of α-SMA immunostaining of a penetrating arteriole in a dsRed mouse, showing α-SMA expression up until the 4th order capillary. On the right are magnifications of the 4th order capillary showing weak but present α-SMA expression of an ensheathing pericyte.*

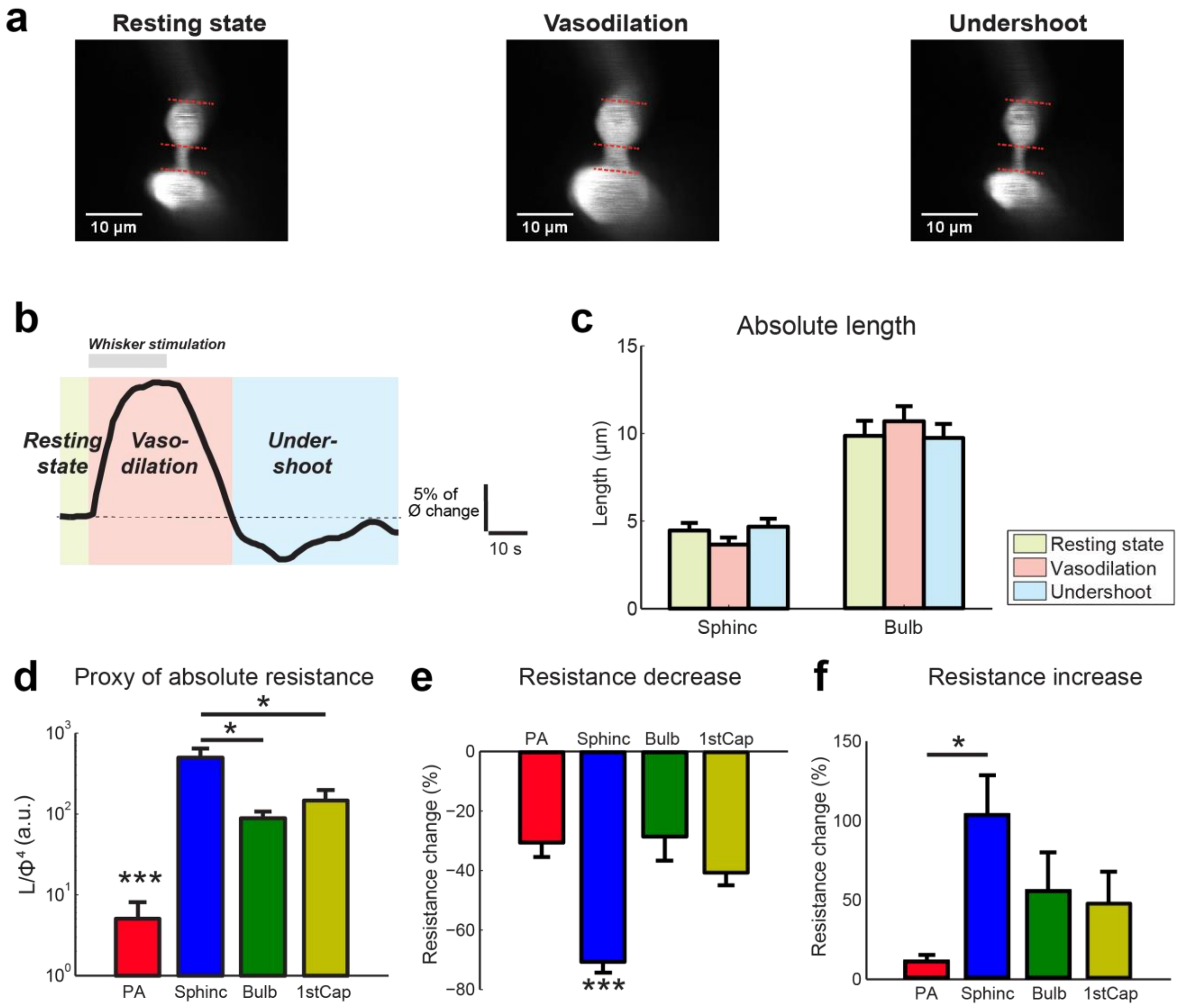

Supplementary Figure 5 | Precapillary sphincter length decreases during stimulation, augmenting the decrease in resistance. ***a***, *Two-photon images of a precapillary sphincter and bulb branching from a PA (lower part). Red lines indicate segment edges of the sphincter and bulb at baseline.* ***b***, *Illustration of the three phases of vasodilation.* ***c***, *Absolute lengths of sphincter and bulb in the three phases of vasodilation.* ***d***, *Poiseuille's law-derived proxy of resistance including the length of the segment at baseline (the length of the PA and 1st order capillary is set to the length of the sphincter).* ***e***, *Decrease in resistance during vasodilation after inclusion of the length change in the proxy of resistance.* ***f***, *Increase in resistance during the undershoot after inclusion of the length change in the proxy of resistance. Source data are provided as a Source Data file.*

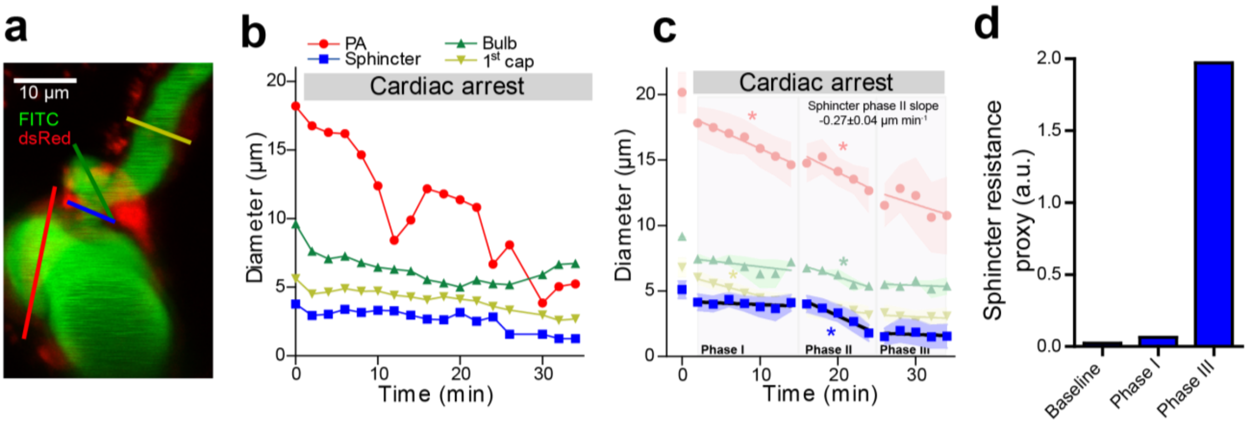

Supplementary Figure 6 | Cardiac arrest and global ischaemia was elicited by intravenous injection of pentobarbital (50 µL of a mix of 200 mg/mL pentobarbital and 20 mg/mL lidocaine) during imaging of the precapillary sphincter. ***a****, Representative maximal intensity projection of an FITC-dextran loaded NG2-dsRed mouse. Colour-coded lines mark the ROIs for repeated diameter measures before and after cardiac arrest.* ***b****, Representative time series of diameter changes at vessel segments during cardiac arrest. The initial diameter decrease is due to the loss of blood pressure.* ***c****, Summary time series of diameter changes after cardiac arrest. Three phases were constructed based on the dynamics of the sphincter. In phase I, the sphincter and bulb diameters held steady, whereas the PA and 1st order capillary diameter gradually declined. After ~15 min (phase II), both the sphincter and bulb start to collapse. After ~25 min (phase III), the sphincter is collapsed and only the PA demonstrates continuing decline. * indicates a slope of the linear regression significantly different from zero, n = 3-7 mice, ±SEM.* ***d****, Estimates of changes in flow resistance within the sphincter using Poiseuille's law at baseline and during phase I and phase III. Source data are provided as a Source Data file.*

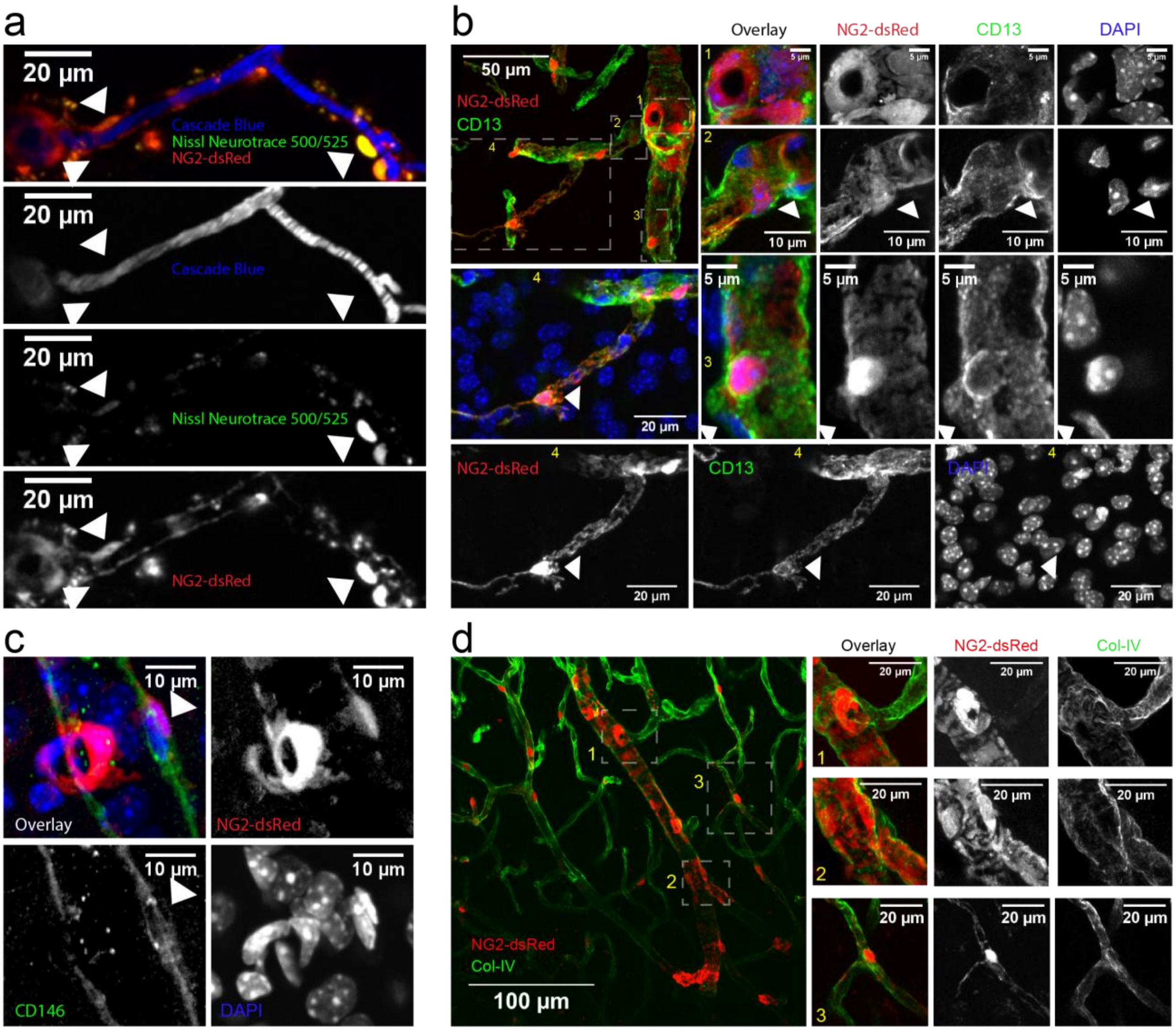

Supplementary Figure 7 | Further molecular characterization of the precapillary sphincter. **a**, Maximum intensity projection image of a PA, precapillary sphincter and 1st-4th order capillary *in vivo* in an NG2-dsRed mouse topically loaded with Nissl neurotrace 500/525. Nissl Neurotrace 500/525 is present in pericytes on >2nd order capillaries, but also to a lower degree in 1st order capillary ensheathing pericytes and to a much lower degree in mural cells on the PA, including the precapillary sphincter (white arrowheads indicate cell bodies). The highest Nissl Neurotrace 500/525 signal was found in pial macrophages (not shown). **b-d**, Maximum intensity projection images of PA, precapillary sphincter and capillaries in *ex vivo* coronal brain slices. **b**, CD13 (Anpep) stained the surface of string/mesh pericytes, but also ensheathing pericytes, the precapillary sphincter and mural cells on the PA. Top left: overview projection of the PA and capillaries with dashed boxes indicating the local projections. Other panels: local projections of precapillary sphincter (1), 1st order capillary with bulb (2), penetrating arteriole (3) and 2nd order capillary with string/mesh pericyte (4). **c**, CD146 was not present at the precapillary sphincter, whereas endothelial cells and to a lesser degree mural cells on the PA showed CD146 signal. **d**, Collagen alpha 1 type IV is a marker of the basement membrane extracellular matrix and stained the outside of all mural cells, including the precapillary sphincter. Left: overview projection of the PA and capillaries with dashed boxes indicating the local projections. Right panels: local projections of a precapillary sphincter and 1st order capillary (1), an arteriole branch without a sphincter (2) and a string pericyte on a high order capillary (3).

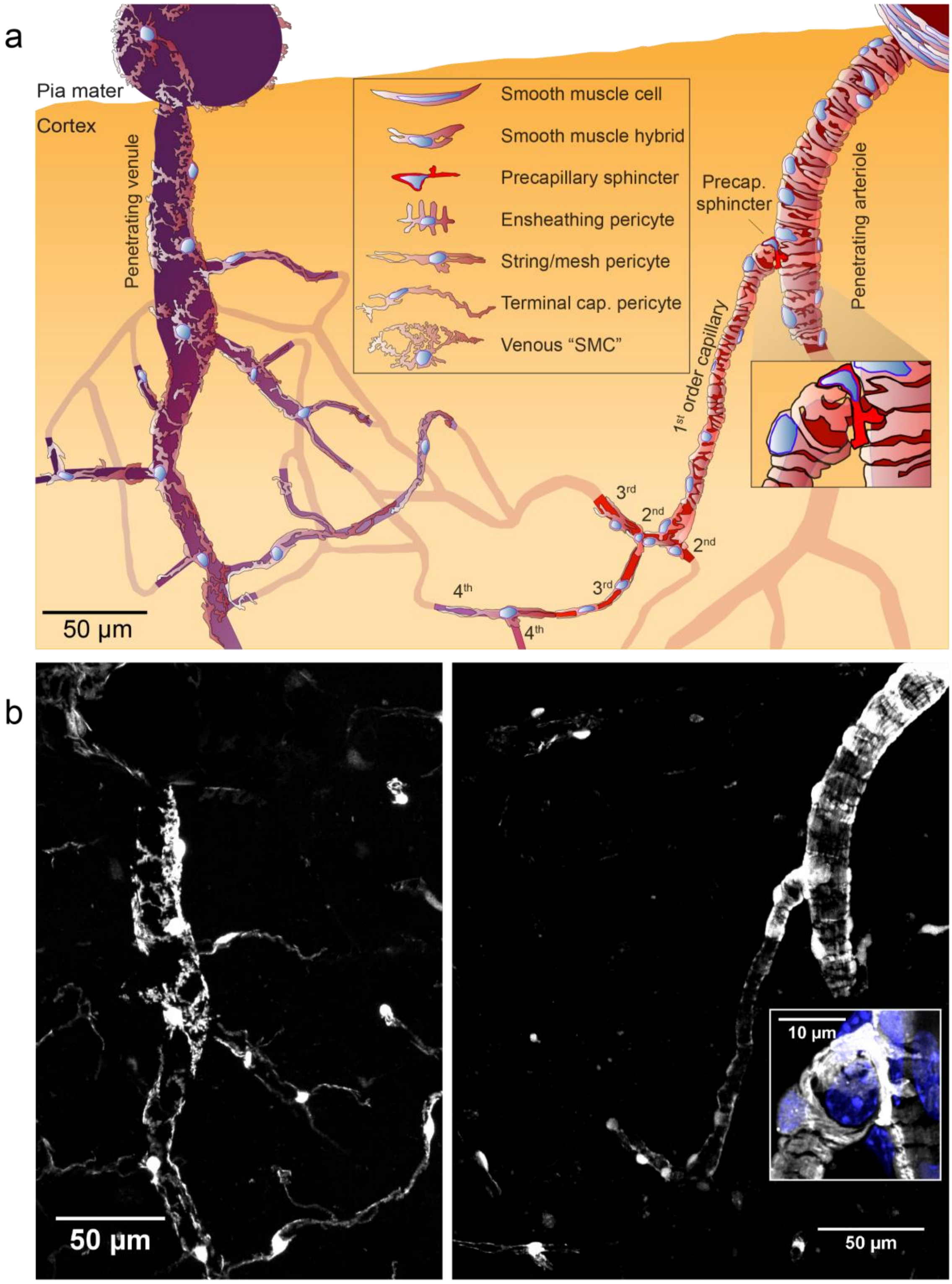

Supplementary Figure 8 | Illustration of the cerebral microcirculation based on confocal images. ***a***, *This illustration show the morphologies of NG2-dsRed positive mural cells from large pial arterioles (>30 µm) via penetrating arterioles to capillaries and via penetrating venules to large pial venules.* ***b***, *The morphologies of the mural cells were drawn as an overlay of two (arteriole and venule) maximal intensity projected high resolution confocal images of DAPI-stained (only shown in high magnification inset) coronal brain slices of NG2-dsRed mice, using the z-stacks as reference.*
