# Supplementary material for: Precapillary sphincters maintain perfusion in the cerebral cortex
Source: Nat Commun. 2020 Jan 20;11:395. doi: 10.1038/s41467-020-14330-z (PMC6971292; doi:10.1038/s41467-020-14330-z)
Supplement: Supplementary file 3 — Description of Additional Supplementary Files [file 41467_2020_14330_MOESM3_ESM.pdf]

Description of Additional Supplementary Files

File Name: Supplementary Movie 1
Description: Thinned skull. Video of a thinned skull experiment, where the focus shifts in 2-µm steps from the skull (notice the NG2-dsRed expression in osteoblasts) through the vasculature in the dura to the pial arterioles. Close-ups of the precapillary sphincters are shown in Supplementary Fig. 2.

File Name: Supplementary Movie 2
Description: Volume rendering of SMA staining. Z-stack and volume rendering of smooth muscle actin antibody and DAPI staining at a precapillary sphincter of an NG2-dsRed mouse brain after i.v. fluorescent-lectin injection.

File Name: Supplementary Movie 3
Description: 4D whisker pad stimulation. Hyperstack projection of precapillary sphincter dilation after whisker pad stimulation. At the end of the video, we show rotation around the x-axis of the baseline and stimulation peak.

File Name: Supplementary Movie 4
Description: RBC stalled at sphincter. Video of RBCs stalling at the precapillary sphincter in an in vivo experiment with a craniotomy (first video) and two pial arteriole precapillary sphincters of a thinned skull experiment (second video).

File Name: Supplementary Movie 5
Description: RBC deformation. Resonance frame-scan video of RBCs passing through a precapillary sphincter and taking up the parachute form. The RBCs move in the opposite direction of the resonance scan, which will flatten their shapes.

File Name: Supplementary Movie 6
Description: CSD. Video of the three phases of the CSD in a dsRed mouse with FITC-dextran in the vessel lumen.

File Name: Supplementary Movie 7
Description: Cardiac arrest. Hyperstack recording of two PA branches with precapillary sphincters in an NG2-dsRed mouse that collapse in rigor after cardiac arrest (first video). The second video shows SR101 staining of astrocytes in an FITC-loaded wild-type mouse in which the astrocytes swell while the vascular lumen collapses.
