# Supplementary material for: Precapillary sphincters maintain perfusion in the cerebral cortex
Source: Nat Commun. 2020 Jan 20;11:395. doi: 10.1038/s41467-020-14330-z (PMC6971292; doi:10.1038/s41467-020-14330-z)
Supplement: Supplementary file 11 — Reporting Summary [file 41467_2020_14330_MOESM11_ESM.pdf]

nature research

Corresponding author(s): Søren Grubb & Martin Lauritzen

Last updated by author(s): Sep 17, 2019

Reporting Summary

Nature Research wishes to improve the reproducibility of the work that we publish. This form provides structure for consistency and transparency in reporting. For further information on Nature Research policies, see Authors & Referees and the Editorial Policy Checklist.

Statistics

For all statistical analyses, confirm that the following items are present in the figure legend, table legend, main text, or Methods section.

n/a	Confirmed	
☐	☒	The exact sample size (*n*) for each experimental group/condition, given as a discrete number and unit of measurement
☐	☒	A statement on whether measurements were taken from distinct samples or whether the same sample was measured repeatedly
☐	☒	The statistical test(s) used AND whether they are one- or two-sided<br>*Only common tests should be described solely by name; describe more complex techniques in the Methods section.*
☒	☐	A description of all covariates tested
☐	☒	A description of any assumptions or corrections, such as tests of normality and adjustment for multiple comparisons
☐	☒	A full description of the statistical parameters including central tendency (e.g. means) or other basic estimates (e.g. regression coefficient) AND variation (e.g. standard deviation) or associated estimates of uncertainty (e.g. confidence intervals)
☐	☒	For null hypothesis testing, the test statistic (e.g. *F*, *t*, *r*) with confidence intervals, effect sizes, degrees of freedom and *P* value noted<br>*Give P values as exact values whenever suitable.*
☒	☐	For Bayesian analysis, information on the choice of priors and Markov chain Monte Carlo settings
☐	☒	For hierarchical and complex designs, identification of the appropriate level for tests and full reporting of outcomes
☒	☐	Estimates of effect sizes (e.g. Cohen's *d*, Pearson's *r*), indicating how they were calculated

Our web collection on statistics for biologists contains articles on many of the points above.

Software and code

Policy information about availability of computer code

Data collection	MATLAB R2016b, Femtonics MES v.6.2.4858, Spike2 v.7.12, Olympus Fluoview FVMPE-R, Zen 2.6
Data analysis	Python 2, Prism 5, RStudio v.1.1.463, ImageJ v.1.51j8, Amira Avizo FEI v.6.5, velocity_from_tif v.02-07-09 (Schaffer Lab)

For manuscripts utilizing custom algorithms or software that are central to the research but not yet described in published literature, software must be made available to editors/reviewers. We strongly encourage code deposition in a community repository (e.g. GitHub). See the Nature Research guidelines for submitting code & software for further information.

Data

Policy information about availability of data

All manuscripts must include a data availability statement. This statement should provide the following information, where applicable:

- Accession codes, unique identifiers, or web links for publicly available datasets
- A list of figures that have associated raw data
- A description of any restrictions on data availability

The data that supports the findings of this study are available from the corresponding author upon request.

Field-specific reporting

Please select the one below that is the best fit for your research. If you are not sure, read the appropriate sections before making your selection.

☒ Life sciences ☐ Behavioural & social sciences ☐ Ecological, evolutionary & environmental sciences

Life sciences study design

All studies must disclose on these points even when the disclosure is negative.

Sample size	We did not predetermine sample size but aimed for a sample of 8 independent mice in each group? Using LME as a statistical tool, we could image across multiple sites in each animal increasing the statistical power of our analysis
Data exclusions	In Figure 3, we excluded RBC velocity measurements from precapillary sphincters where there were no diameter changes in response to whisker stimulation, or where there was no precapillary sphincter. Otherwise, we did not exclude data from the analysis.
Replication	We used a sample size (independent animals) of at least 8 per sub-study. In different sub-studies, the same sample size applied, effectively adding the total sample size if the same characteristic was under observation
Randomization	In this observational study we did not assess the effect of treatments but rather the different behavior of vessel segments to various treatments. Therefore randomization was not applicable to our study.
Blinding	We did not have blinding in our experiments. First, this is an observational study. Second, we used a particular procedure to induce stimulation, CSD, or cardiac arrest that did not lend to proper blinding. As we did not look at the effect of different treatments (but rather vessel segment differences) blinding was also not applicable to data analyses.

Reporting for specific materials, systems and methods

We require information from authors about some types of materials, experimental systems and methods used in many studies. Here, indicate whether each material, system or method listed is relevant to your study. If you are not sure if a list item applies to your research, read the appropriate section before selecting a response.

Materials & experimental systems

n/a	Involved in the study	
☐	☒	Antibodies
☒	☐	Eukaryotic cell lines
☒	☐	Palaeontology
☐	☒	Animals and other organisms
☒	☐	Human research participants
☒	☐	Clinical data

Methods

n/a	Involved in the study	
☒	☐	ChIP-seq
☒	☐	Flow cytometry
☒	☐	MRI-based neuroimaging

Antibodies

Antibodies used

Primary Antibodies: mouse ACTA2-FITC (1:200; Sigma; F3777), Rabbit anti-collagen-I (1:50; ab34710), Mouse Vitronectin antibody (1:100, R & D systems, #347317), Goat Aminopeptidase N/CD13 Antibody (1:100, R & D Systems, AF2335), Rat MCAM/CD146 Antibody (1:100, R & D Systems, MAB7718), Rabbit Anti-Collagen Antibody, Type IV (1:100, Merck Millipore, AB756P).
Secondary antibody: Rabbit Alexa488 (1:500, ThermoFisher SCIENTIFIC), Mouse Alexa488 (1:500, ThermoFisher SCIENTIFIC), Rat Alexa488 (1:500, ThermoFisher SCIENTIFIC), Goat Alexa488 (1:500, ThermoFisher SCIENTIFIC).

Validation

Primary Antibodies:
mouse ACTA2-FITC (1:200; Sigma; F3777).
Details: Monoclonal Anti-Actin, Smooth Muscle-FITC, clone 1A4
Produced in mouse, purified immunoglobulin, FITC conjugate
Source: mouse
Isotype: IgG2a
Tested application: immunohistochemistry
Validation Species: rabbit, guinea pig, mouse, chicken, snake, sheep, goat, human, frog, rat, canine, bovine
UniProt accession no: P62736
References:
https://www.sigmaaldrich.com/catalog/product/sigma/f3777?lang=en&region=DK
https://www.sigmaaldrich.com/catalog/papers/8698823

Rabbit anti-collagen-I (1:50; ab34710).
Rabbit polyclonal to Collagen I
Source: Rabbit
Isotype: IgG
Tested application: Immunohistochemistry, Immunocytochemistry, ELISA, Western Blot
Validation species: Mouse, rat, sheep, goat, horse, cow, human, pig, common marmoset
Database link: P02452
References:
• Yan Y et al. Vascularized 3D printed scaffolds for promoting bone regeneration. Biomaterials190-191:97-110 (2019).

• Wang D et al. Insulin-like growth factor-1 engaged in the mandibular condylar cartilage degeneration induced by experimental unilateral anterior crossbite. Arch Oral Biol 98:17-25 (2019).

Rat Vitronectin antibody (1:100, R & D systems, #347317)
Details: Monoclonal Rat IgG2A, Clone #347317
Source: Rat
Isotype: IgG2a
Tested application: immunohistochemistry Validation species: mouse
References:
https://www.rndsystems.com/products/mouse-vitronectin-antibody-347317_mab38751
He L, Vanlandewijck M, Raschperger E, Andaloussi Mäe M, Jung B, Lebouvier T, Ando K, Hofmann J, Keller A, Betsholtz C. 2016. Analysis of the brain mural cell transcriptome. Sci Rep. 6:35108. d Franco M, Roswall P, Cortez E, Hanahan D, Pietras K. 2011. Pericytes promote endothelial cell survival through induction of autocrine VEGF-A signaling and Bcl-w expression. Blood 118:2906-2917.

Goat Aminopeptidase N/CD13 Antibody (1:100, R & D Systems, AF2335)
Details: Antigen Affinity-purified Polyclonal Goat IgG, Catalog Number: AF2335
Source: goat
Isotype: IgG
Tested application: Immunohistochemistry Validation species: mouse
References:
https://www.rndsystems.com/products/mouse-aminopeptidase-n-cd13-antibody_af2335
He L, Vanlandewijck M, Raschperger E, Andaloussi Mäe M, Jung B, Lebouvier T, Ando K, Hofmann J, Keller A, Betsholtz C. 2016. Analysis of the brain mural cell transcriptome. Sci Rep. 6:35108.

Rat MCAM/CD146 Antibody (1:100, R & D Systems, MAB7718)
Details: Monoclonal Rat IgG2A Clone #733216
Source: Rat
Isotype: IgG
Tested application: immunohistochemistry in mouse cell line
References: https://www.rndsystems.com/products/mouse-mcam-cd146-antibody-733216_mab7718

Rabbit Anti-Collagen Antibody, Type IV (1:100, Merck Millipore, AB756P)
Details: Polyclonal to Collagen alpha 1 Type IV (LOT: 2972819)
Source: Rabbit
Isotype: IgG
Tested application: immunohistochemistry
References:
http://www.merckmillipore.com/DK/en/product/Anti-Collagen-Antibody-Type-IV,MM_NF-AB756P

Secondary antibody:
Rabbit Alexa488 (1:500, ThermoFisher SCIENTIFIC)
Source: goat
Isotype: Goat IgG
Tested application: Flow cytometry, Immunohistochemistry, Immunocytochemistry, CHIP assay, Western Blot
Validation species: rabbit
References:
https://www.thermofisher.com/antibody/product/Goat-anti-Rabbit-IgG-H-L-Cross-Adsorbed-Secondary-Antibody-Polyclonal/A-11008
Recombinant Adeno-Associated Virus Serotype 6 (rAAV6) Potently and Preferentially Transduces Rat Astrocytes In vitro and In vivo. Schober AL,Gagarkin DA,Chen Y,Gao G,Jacobson L,Mongin AA

Mouse Alexa488 (1:500, ThermoFisher SCIENTIFIC)
Source: Goat
Isotype: Goat IgG
Validation species: Mouse

Rat Alexa488 (1:500, ThermoFisher SCIENTIFIC)
Source: Rabbit
Isotype: Rabbit IgG
Validation species: Rat

Goat Alexa488 (1:500, ThermoFisher SCIENTIFIC)
Source: Chicken
Isotype: Chicken IgG
Validation species: Goat

Animals and other organisms

Policy information about studies involving animals; ARRIVE guidelines recommended for reporting animal research

Laboratory animals	A total of 38 male or female NG2-dsRed mice (Tg(Cspg4-DsRed.T1)1Akik/J; Jackson Laboratory; 19 to 60 weeks old) and 27 male or female wild-type mice (C57bl/6j; Janvier-labs, France;16 to 32 week) were used.

Wild animals	The study did not involve wild animals
Field-collected samples	The study did not involve samples collected from the field
Ethics oversight	Animal procedures were approved by The Danish National Ethics committee according to the guidelines set forth in the European Council's Convention for the Protection of Vertebrate Animals Used for Experimental and Other Scientific Purposes.

Note that full information on the approval of the study protocol must also be provided in the manuscript.
